# Supplementary material for: Metal Ions Modify In Vitro DNA Damage Yields with High-LET Radiation
Source: Toxics. 2023 Sep 12;11(9):773. doi: 10.3390/toxics11090773 (PMC10537317; doi:10.3390/toxics11090773)
Supplement: Supplementary file 1 [file toxics-11-00773-s001.zip › toxics-2593697-supplementary.pdf]

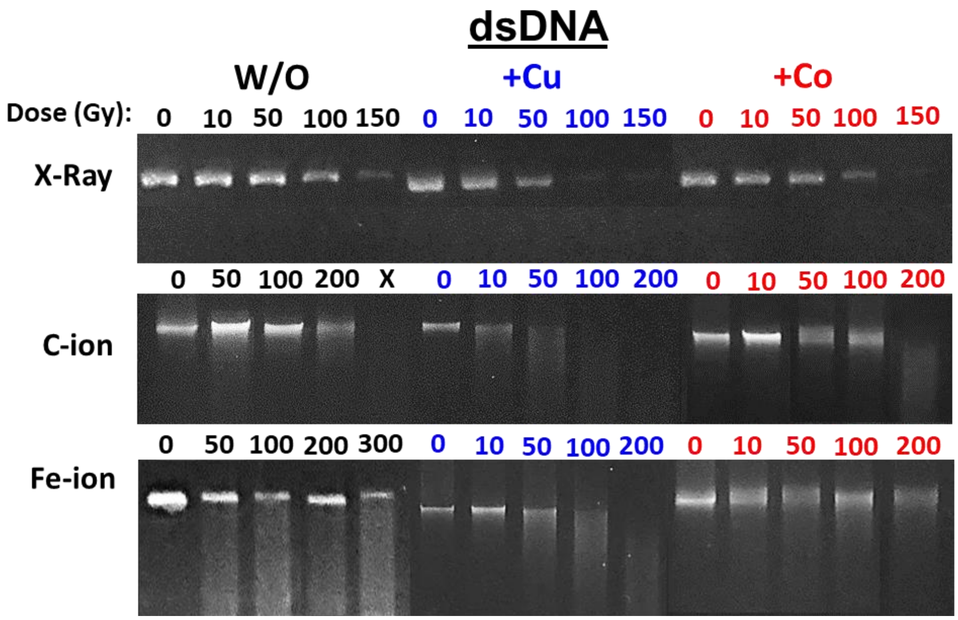

Figure S1. dsDNA Agarose gel images depicting the amount of dsDNA following each radiation source at increasing irradiation dosages of solutions without metal ions (w/o), with Cu^{2+} (+Cu) or with Co^{2+} (+Co)

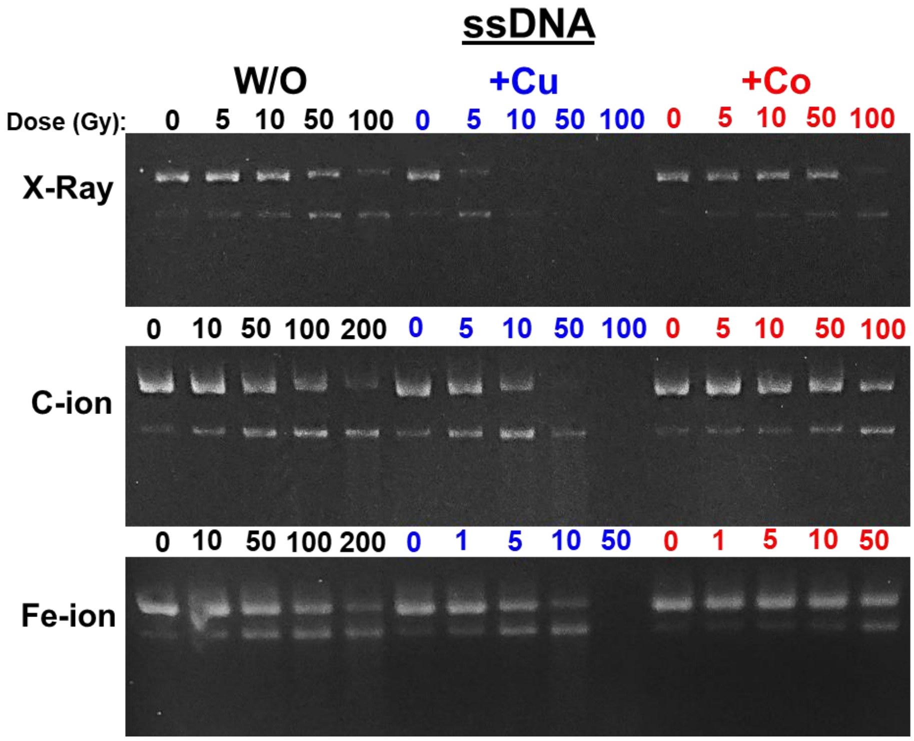

Figure S2. ssDNA Agarose gel images depicting the amount of ssDNA following each radiation source at increasing irradiation dosages of solutions without metal ions (w/o), with Cu^{2+} (+Cu) or with Co^{2+} (+Co)

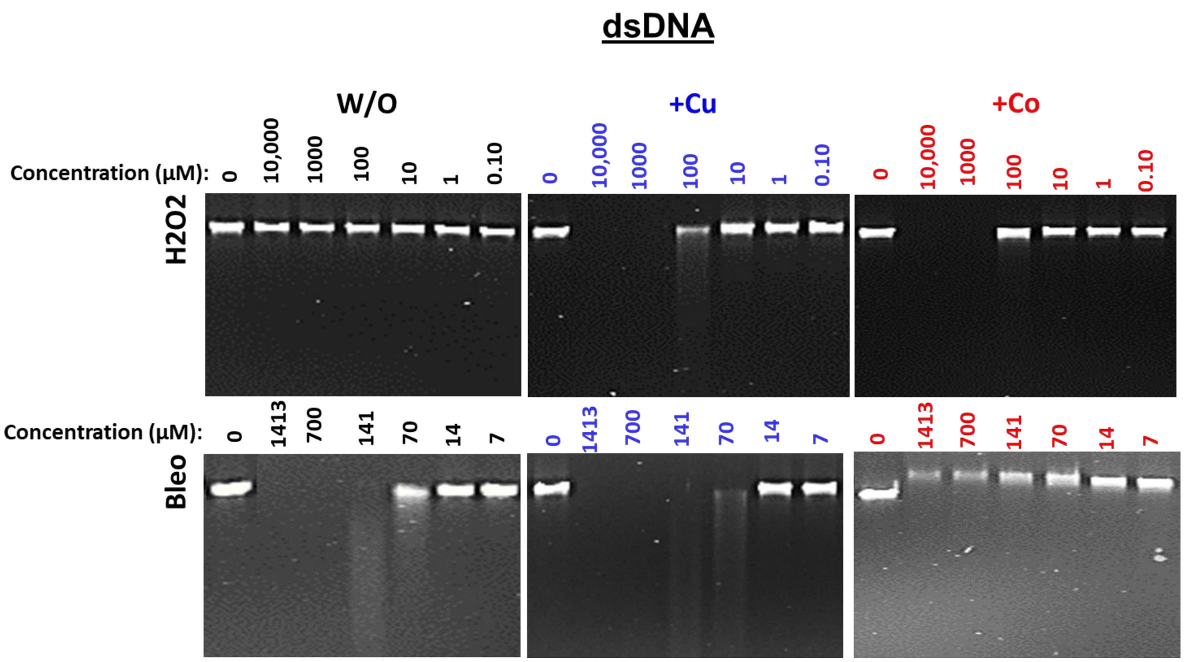

Figure S3. dsDNA Agarose gel images depicting the amount of dsDNA following chemical treatment (H_2O_2 or Bleomycin) at increasing chemical concentrations in solutions without metal ions (w/o), with Cu^{2+} (+Cu) or with Co^{2+} (+Co)

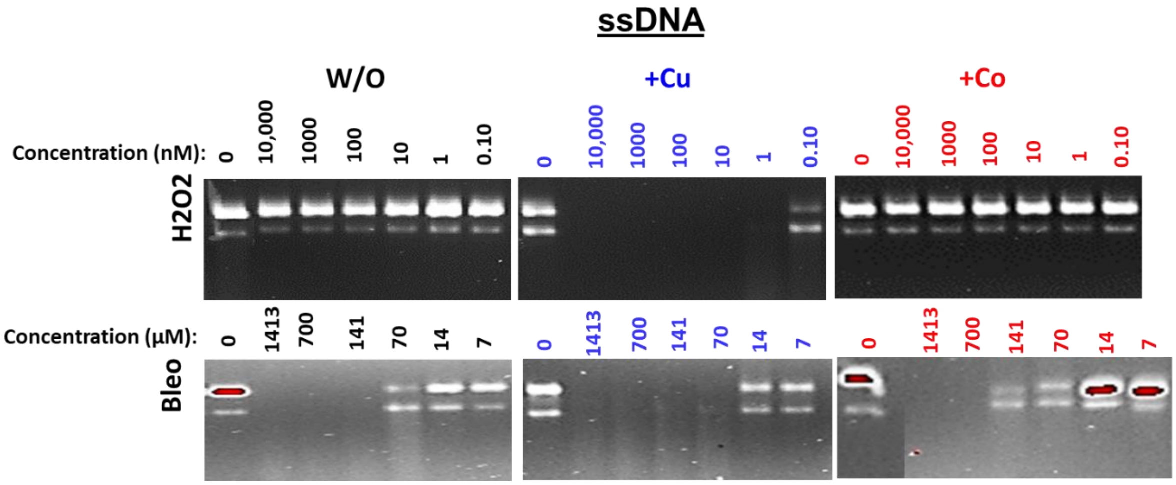

Figure S4. ssDNA Agarose gel images depicting the amount of ssDNA following chemical treatment (H_2O_2 or Bleomycin) at increasing chemical concentrations in solutions without metal ions (w/o), with Cu^{2+} (+Cu) or with Co^{2+} (+Co)
